# PD28 Characteristics And Outcomes Of Blood Cell Saver Use In Scoliosis Surgery: A Brazilian Health Maintenance Organization Analysis

**DOI:** 10.1017/S0266462325101633

**Published:** 2025-12-29

**Authors:** Maria da Glória Cruvinel Horta, Marcus Borin, Mariana Michel Barbosa, Carina Rejane Martins, Daniel Pitchon dos Reis, Geraldo José Coelho Ribeiro, Júlia Teixeira Tupinambás, Karina de Castro Zocrato, Lélia Maria de Almeida Carvalho, Marcela Pinto de Freitas, Mariza Cristina Torres Talim, Sergio Adriano Loureiro Bersan, Silvana Marcia Bruschi Kelles

## Abstract

**Introduction:**

Severe scoliosis affects three percent of the population, with 0.3 percent requiring surgery that often results in significant blood loss. The cell saver technique collects and reinfuses lost blood, potentially reducing the need for transfusions. This study evaluated its clinical outcomes and feasibility in scoliosis surgeries performed between January 2022 and March 2024 in a Brazilian Health Maintenance Organization.

**Methods:**

A non-concurrent cohort study analyzed the use of cell savers in scoliosis surgeries performed between January 2022 and March 2024 within a non-profit health assistance organization in Brazil. Patients were divided into two groups: those who underwent intraoperative use of the cell saver technique and those who did not. Data on patient demographics, procedural details, and clinical outcomes were collected from institutional databases. Primary outcomes included the length of hospital stay, the need for blood transfusion, and rates of major complications. Statistical comparisons were performed using chi-square tests and t-tests (statistical significance set at p<0.05).

**Results:**

Among 311 patients, nine received the cell saver technique. The mean age was 14.8 years, with 66 percent being younger than 19 years. In the non-cell saver group (302 patients), 36 percent required blood transfusions (average two units/patient) and the mean hospital stay was 7.52 days. In the cell saver group, 44 percent required transfusions (average 1.25 units/patient), and the mean hospital stay was 6.89 days. The results suggested trends toward reduced transfusion needs, use of fewer blood units, and shorter hospital stays in the cell saver group, although the sample size was limited.

**Conclusions:**

This study identified potential benefits of intraoperative cell saver use in scoliosis surgeries, including reduced transfusion needs, use of fewer blood units, and shorter hospital stays. However, the findings require validation in larger, more diverse samples to establish clinical significance and cost effectiveness in routine practice.

